# Assessing oral health-related quality of life among patients with oral submucous fibrosis

**DOI:** 10.6026/973206300211467

**Published:** 2025-06-30

**Authors:** Pingal Chandrashekhar Shriram, Saurabh Kothari, Himani Marmat, Subasish Behera, Dakshayani Vijay Patil, Soumyaranjan Nanda, Miral Mehta

**Affiliations:** 1 Department of Oral and Maxillofacial Surgery, College of Dental Science & Hospital, Amargadh, Bhavnagar, Gujarat, India; 2 Department of Public Health Dentistry, Jaipur Dental College, Jaipur, Rajasthan, India; 3 Department of Medicine, MGM and M.Y. Superspeciality Hospital, Indore, Madhya Pradesh, India; 4 Department of Conservative Dentistry and Endodontics, Government Dental College and Hospital (SCB), Manglabag, Cuttack, Odisha, India; 5 Department of Oral Pathology and Microbiology, Hazaribag College of Dental Sciences and Hospital, Jharkhand, India; 6 Endodontics, Private Practitioner, Cuttack, Odisha, India; 7 Department of Pediatric and Preventive Dentistry, Karnavati School of Dentistry, Karnavati University, Gandhinagar, Gujarat, India

**Keywords:** Oral submucous fibrosis, quality of life, OHIP-14, oral health, mouth opening, OHRQoL, areca nut

## Abstract

Oral submucous fibrosis (OSMF) is chronic and can be cancerous, causing difficulties with using the mouth. Therefore, it is of interest evaluate the oral health-related quality of life (OHRQoL) in 120 OSMF patients by using the OHIP-14 scale. Restricted mouth opening was associated with worse OHIP-14 scores in patients (p < 0.001). Problems with activity and mental health were the biggest changes measured. The report points out that early detection and treatment help improve the quality of life in OSMF sufferers.

## Background:

Chronic and serious Oral Submucous Fibrosis mainly affects the mouth, strongly preferring the inner lining of the cheeks, the rear top part of the mouth and the oropharynx. On microscopic analysis of the disease, changes include reduction of glands, inflammation just below the glands and increased mucosal fibrosis, leading to gradual difficulty in opening the mouth because of stiffening [1]. Ocular surface dysfunction was first identified in the early 1950s and has later grown into a serious concern for public health in South and Southeast Asia, affecting mainly India, Taiwan, Pakistan and Sri Lanka [2]. Being a potentially malignant condition, OSMF is important in medical care since about 7-13% of these patients may develop oral squamous cell carcinoma [3, 4]. Being chewed daily, often with tobacco, is the main cause of OSMF and advanced use of areca nut also plays a role. Other reasons for indigestion may be nutrient shortages, extra irritation due to frequent spicy foods, susceptibility in certain groups and problems with the immune response [5, 6]. Because areca nut is a Group 1 carcinogen, the chemicals in it, including arecoline and tannins, help increase fibroblast growth and collagen synthesis, causing the submucosal fibrosis found with OCPS [7]. The number of people affected by OSMF is large in India. The rate is usually between 0.2% and 6.3%, but in regular users of gutkha and pan masala, it can go as high as 20%. Although males in the second to fourth decade of life are affected most often, reports reveal that the rise in chewing arecas by adolescents and females is significant [8, 9]. Commonly in the clinic, patients with OSMF have a sore, painful mouth, pale oral tissues, stiff cheeks and growing difficulty opening their mouth-a condition called trismus. When a patient has these symptoms, it affects their ability to chew, swallow, speak and keep their mouth healthy. In later stages, the illness takes away from patient comfort, regular meals and enjoyable social activities, which impact nutrition and mental health [10, 11]. OSMF being a chronic and severely limiting condition, we must also look at its influence. What matters most is how people feel about and deal with their mouth health in everyday situations and social life [12]. One instrument validated globally, OHIP-14, can be used to measure OHRQoL by looking at four aspects, such as difficulties in performing, physical pain, mental health and social participation [13]. By using it in treating chronic oral problems, clinicians gain a patient-oriented framework that goes well with the usual ways of diagnosing and treating such conditions. Researchers have studied OSMF's clinical and pathological factors, but little literature looks at the quality of life of these patients, focusing on Indians in particular. Learning how disease severity affects how people perceive their daily life can help in planning the right counseling programs and strategies. If quality-of-life evaluations are included early on, they can affect resource planning, improve how much treatment is taken and mark success for the therapy [14, 15]. Therefore, it is of interest evaluate the oral health-related quality of life (OHRQoL) in 120 OSMF patients by using the OHIP-14 scale.

## Materials and Methods:

A total of 120 patients clinically diagnosed with Oral Submucous Fibrosis (OSMF) were enrolled using a convenience sampling method. Inclusion criteria consisted of patients aged between 20 and 60 years with a confirmed diagnosis of OSMF, who provided written informed consent. Patients with a history of systemic illness, other oral potentially malignant disorders, malignancy, or those undergoing any treatment for OSMF were excluded from the study. Demographic data, including age, gender, occupation and duration of habits (such as areca nut and tobacco use), were recorded through a structured interview. Clinical examination was conducted to assess mouth opening using a vernier caliper and participants were categorized into three groups based on interincisal distance:

[1] Group I: Mouth opening >35 mm

[2] Group II: Mouth opening between 20-35 mm

[3] Group III: Mouth opening <20 mm

Analysis of oral health-related quality of life involved applying the Oral Health Impact Profile-14 (OHIP-14) test. The tool is used to find out the frequency of problems in seven areas: functional limitation, physical pain, psychological discomfort, physical disability, psychological disability, social disability and handicap. The assessment used a 5-point scale that ran from 0 (never) to 4 (very often) for every item. The scale scores were between 0 and 56 and individuals who scored higher had worse oral health-related quality of life. All data were loaded into Microsoft Excel and analyzed with SPSS version 25.0, made by IBM Corp (Armonk, NY, USA). We used the mean and standard deviation for continuous variables and showed categorical ones as frequencies and percentage figures. A one-way ANOVA was performed on the OHIP-14 difference scores to find out if they varied between the groups and a Tukey's test was used afterward to compare all pairs of groups. For results to be significant, a p-value had to be less than 0.05.

## Results:

A total of 120 patients diagnosed with Oral Submucous Fibrosis were included in the study, comprising 76 males (63.3%) and 44 females (36.7%). The mean age of participants was 38.4 ± 9.7 years. Patients were grouped based on interincisal mouth opening: Group I (>35 mm) included 34 patients (28.3%), Group II (20-35 mm) had 48 patients (40%) and Group III (<20 mm) comprised 38 patients (31.7%). The mean OHIP-14 scores for each group are summarized in Table 1 (see PDF). Patients in Group III, with the most severe restriction of mouth opening, demonstrated the highest OHIP-14 score (mean = 29.1 ± 5.1), indicating a significantly impaired quality of life. In contrast, Group I showed the lowest mean score (18.3 ± 3.9), suggestive of comparatively better oral health-related quality of life. The overall mean OHIP-14 score across all participants was 23.5 ± 4.8. A statistically significant difference in OHIP-14 scores was observed among the three groups (p < 0.001) (Table 1 - see PDF). The domain-wise distribution of OHIP-14 responses is presented in Table 2 (see PDF). Physical pain (mean = 4.9 ± 1.2) and functional limitation (mean = 4.6 ± 1.3) were the most severely affected domains across all participants, followed by psychological discomfort (mean = 4.2 ± 1.5). Social disability and handicap domains recorded the lowest impact scores (mean = 2.7 ± 1.1 and 2.3 ± 0.9, respectively) (Table 2 - see PDF). Post hoc analysis using Tukey's test confirmed that differences between Group I and Group III and between Group II and Group III, were statistically significant (p < 0.01), while the difference between Group I and Group II was also significant, though less pronounced (p = 0.04). These results indicate a clear inverse relationship between the degree of mouth opening and the perceived oral health-related quality of life among patients with OSMF.

## Discussion:

OSMF is a problem in the mouth that impacts a person's ability to eat, speak clearly and maintain oral health. Researchers measured the oral health-related quality of life of OSMF patients by using the OHIP-14 questionnaire. Reduced mouth opening resulting from OSMF was linked by this study to significant loss of quality of life, mainly in terms of pain and limitation in daily activities. Our study is in line with others that have noted that OSMF may block movement in the mouth and lead to large problems in social and psychological health [1, 2]. Similar to earlier studies in Indian regions with high cases [3, 4], we found that the mean OHIP-14 score was 23.5 ± 4.8. Because the buccal mucosa and soft palate of OSMF thicken, individuals start to have trouble chewing and swallowing food, which results in under nutrition, a drop in body weight and distress [5, 6]. Our study showed that both functional difficulties and pain stood out in terms of impact, just as Alora Veedu *et al.* pointed out that burning discomfort and stiffening of mouth tissues often negatively affect oral movements [7]. Because of the tightening of the mouth, people with OSMF are unable to eat solid foods, which often cause them to feel ashamed and separate themselves from others [8]. Group III patients (<20 mm mouth opening) had the highest OHIP-14 scores, suggesting that a severe case of trismus leads to a significant decline in OHRQoL, as Ranganathan and his colleagues found earlier [9]. OSMF also has a strong effect on people's emotional well-being. The testing discovered a high range of psychological discomfort and psychological disability in our population. Living with the risk of cancer, continued pain and poor speech skills causes many of these patients to feel more anxious and depressed [10, 11]. Psychological stress found in emotional abuse can worsen how pain is perceived and lower the patient's compliance with the treatment [12].

We need to address OSMF as a whole body condition, not only as a problem with the mouth structure. Our study benefited from including the OHIP-14 tool, which helped show the effects of OSMF from multiple sides. According to Adhikari *et al.* OHRQoL instruments should be applied routinely in practice to improve how patients are cared for [13]. Our study shows that this is true, as people with very serious diseases may look stable, but still feel severely distressed. We should note that, even with the wide prevalence of OSMF, information about the condition and its issues is not common among many individuals and numerous health professionals. Complications emerge in many cases when patients cannot get early diagnosis and advice, which adds greater problems for them [14, 15]. Local education programs and early screenings might significantly help slow the disease and its impact on individuals and families [16, 17-18]. The study is limited because it only involved a single data collection point, so we cannot determine whether the treatment really caused effects. Since people were enrolled using only a single center and were sampled conveniently, the results may not apply to the general population. But when OHIP-14 is applied, the measured outcomes show greater reliability.

## Conclusion:

OSMF reduces oral health-related quality of life, mainly in people with serious mouth-opening limitations. The findings make it clear why early diagnosis, information sharing with patients and targeting all aspects of the disease are essential.
